# Unwrapping Bacteria

**DOI:** 10.1371/journal.pgen.1004054

**Published:** 2014-01-02

**Authors:** Kevin D. Young

**Affiliations:** Department of Microbiology and Immunology, University of Arkansas for Medical Sciences, Little Rock, Arkansas, United States of America; Indiana University, United States of America

Imagine you must wrap a present—from the inside. You (as the “gift”) must first cover yourself with one or more sheets of light tissue paper, to keep from getting smudged or rattling around. Next, without leaving or tearing this papery cocoon, you must construct a rugged box to encase both gift and tissue, to protect against mishaps and external onslaughts. Finally, from deep within, using no tools that would damage or mar these previous bits of handiwork, you must overlay the whole parcel with a thin film of wrapping paper, colorfully patterned on its outer side but plain on its inner. If you are even more exuberant you may add ribbons, bows, and baubles to spruce up the completed package, but all the while you must remain embedded at center of these nested shells.

Pretty impressive trick, huh?

**Figure 1:**
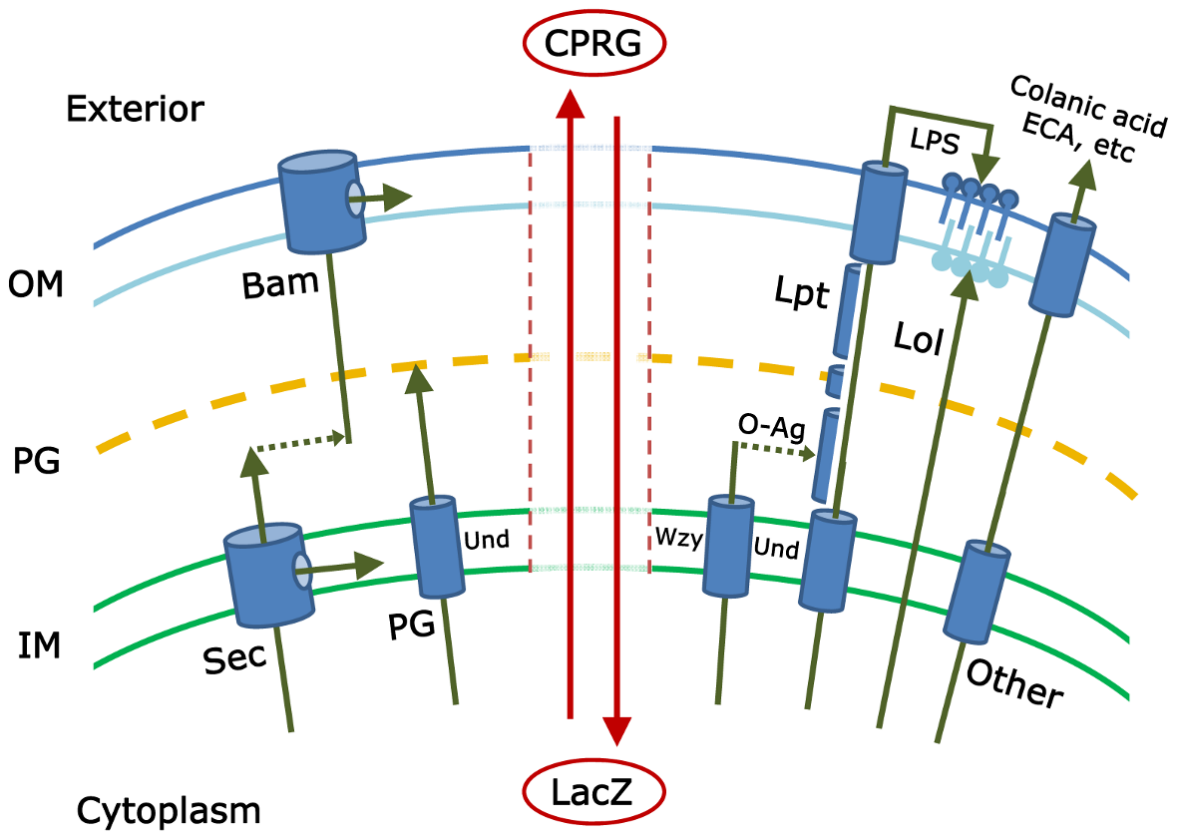
This schematic illustrates *some* of the pathways that must be coordinated to create an intact gram-negative bacterial cell envelope. The inner membrane (IM), peptidoglycan (PG), and outer membrane (OM) form the principal layers into which components are inserted by pathways referenced in the text. The isoprenoid compound undecaprenyl-phosphate (Und) is an integral part of several of these pathways. The vertical central zone represents envelope damage that may accompany mutations in these pathways; breakdown of envelope integrity may allow the cytoplasmic LacZ protein to come into contact with and hydrolyze the extracellular compound CPRG (chlorophenyl red-β-D-galactopyranoside). The resulting red color marks the colonies of such mutants and is the basis for the genetic screen described by Paradis-Bleau et al. Note: the actual gram-negative envelope is much more complicated and includes, for example, the Tat secretory pathway; other specialized protein secretion systems; and additional envelope proteins, extracellular components, and pathways. Any of these may contribute to envelope integrity and therefore may be subjects of study by the screen introduced by Paradis-Bleau et al. [1].

This lighthearted metaphor describes something very close to what gram-negative bacteria do on a moment-to-moment basis as they create the envelope that surrounds and defends them. The cytoplasm (the “gift”) is surrounded by the inner membrane (the “tissue paper”), the peptidoglycan cell wall (the “box”), and the outer membrane (the “wrapping paper”) (see Figure 1). In truth, the task is even more difficult than suggested by this analogy because the components must grow as the cell grows, divide when the mass doubles, allow some materials to cross while excluding others, and protect against a high internal turgor pressure, and none of these activities must compromise the integrity of any other element. In short, to create a cell “from the inside” requires the coordination of a remarkable suite of strategies and competing biochemical reactions. How all this is accomplished is the core concern of a new technique introduced by Paradis-Bleau et al. in this issue of *PLOS Genetics* [1].

To date, investigators have pieced together the major mechanisms by which each envelope subcomponent is synthesized and directed to one of four destinations (inner membrane, periplasm, or to one of the two faces of the outer membrane) (see Figure 1). Proteins are directed to the inner membrane and periplasm (the space between the inner and outer membranes) via the Sec or Tat secretory pathways [2], [3]; lipopolysaccharides and lipoproteins are directed to the outer and inner leaflets of the outer membrane via the Lpt and Lol pathways, respectively [4], [5]; proteins are inserted into the outer membrane by way of the Bam pathway [5]–[7]; the cell wall peptidoglycan is polymerized in the periplasm [8]; and carbohydrate polymers, such as colanic acid, enterobacterial common antigen (ECA), or capsule, are delivered to the cell surface or extracellular space [9]–[11]. And yet, despite all we know, at least two large questions remain. First, have all the pertinent biochemical pathways been described? Probably not, because little or nothing is known about the function of nearly one-third of the ∼400 predicted envelope proteins in *Escherichia coli* [12]. The second, more mysterious and difficult question is this: how are all these pathways choreographed so that the cell grows smoothly? The identities of these crucial regulatory processes are deeply enigmatic.

Paradis-Bleau and colleagues describe a genetic approach that promises to move us closer to finding the answers to these lingering questions [1]. They begin by assuming that mutations in individual biochemical steps or in overarching regulatory pathways will produce cells with defective envelopes. The simplest phenotype of such an envelope is that it becomes porous to substances that are otherwise contained within the cell or excluded from it. Alternately, envelope function may be so severely impaired that a fraction of the bacterial culture dies and releases cytoplasmic material. Paradis-Bleau et al. devised a simple screen to detect mutants that meet either of these criteria. The small molecule CPRG (chlorophenyl red-β-D-galactopyranoside) is incorporated into an agar plate, onto which mutagenized bacteria are spread. Wild-type *E. coli* cells form white colonies on plates containing this compound because CPRG cannot enter intact bacteria. However, cells with an impaired envelope may admit CPRG to the cytoplasm, where the LacZ protein hydrolyzes it to form a red product. Alternatively, if the mutants lyse, LacZ is released into the medium to contact CPRG (see Figure 1). In either case the colonies become visibly red and can be isolated for further study.

When Paradis-Bleau et al. tested a library of random transposon insertions and an ordered set of gene deletions in *E. coli*, the screen identified envelope-damaging effects caused by ∼100 genes with no known function. For example, mutants lacking a functional *elyC* (*ycbC*) gene lysed as they approached stationary phase, a transition point whose regulation is poorly understood. This phenotype was suppressed by overexpressing enzymes involved in the synthesis of peptidoglycan or undecaprenyl pyrophosphate, this latter a critical isoprenoid that participates in the biosynthesis of several envelope components [10], [13], [14]. In addition, mutations in the ECA synthetic pathway exacerbated or suppressed this *elyC*-associated lethality. Thus, characterizing the behavior and genetic interactions of this single mutation has already identified a web of new envelope relationships. More are expected, since the technique allows investigators to bring a whole pantheon of genetic tools to bear on these questions, and because the screen can be adapted to organisms whose envelopes are even less well understood.

 Of course, even such a significant advance does not solve all problems. First, the technique identifies only those mutants that are viable and that disable the envelope so that it leaks. Mutations that kill cells outright will not appear, nor will mutations that alter assembly without affecting envelope permeability. Second, the screen will probably return mutants that die or leak even if the affected processes have no direct bearing on the envelope (though these may be interesting in their own right). Third, there is an odd temperature limitation: the screen works well at 25°C, but a large background appears when cells are incubated at 37°C. The reason is not clear, but this, too, might be turned into a positive by inverting the approach to look for mutants that do *not* leak under these or other conditions, thereby expanding the screen's genetic reach. Thus, even its limitations presage the expansion of the technique for use in broader contexts.

In short, Paradis-Bleau et al. have performed a valuable service by developing this new tool for investigating the complexities of the bacterial cell envelope. And that's a gift for all of us, wrapped just right.
